# *Riemerella anatipestifer* AS87_RS09170 gene is responsible for biotin synthesis, bacterial morphology and virulence

**DOI:** 10.1038/s41598-018-32905-1

**Published:** 2018-10-02

**Authors:** Xiaomei Ren, Xiaolan Wang, Huoying Shi, Xuemei Zhang, Zongchao Chen, Kanwar Kumar Malhi, Chan Ding, Shengqing Yu

**Affiliations:** 1 Shanghai Veterinary Research Institute, Chinese Academy of Agricultural Sciences (CAAS), Shanghai, China; 2 College of Veterinary Medicine, Yangzhou University, Yangzhou, Jiangsu China; 3 Jiangsu Co-innovation Center for Prevention and Control of Important Animal Infectious Diseases and Zoonosis, Yangzhou, Jiangsu China

## Abstract

*Riemerella anatipestifer* is a bacterial pathogen responsible for major economic losses within the duck industry. Recent studies have revealed that biotin biosynthesis is critical for the bacterium’s survival and virulence. We previously found that *R*. *anatipestifer* AS87_RS09170, a putative *bioF* gene, is important for bacterial virulence. In the present study, we characterized the AS87_RS09170 gene in *R*. *anatipestifer* strain Yb2. Sequence analysis indicated that the AS87_RS09170 gene is highly conserved among *R*. *anatipestifer* strains; the deduced protein harbored the conserved pyridoxal 5′-phosphate binding pocket of 8-amino-7-oxononanoate synthase. Western blot analysis demonstrated that the biotin-dependent enzyme was present in smaller quantities in the mutant strain Yb2ΔbioF compared to that of the wide-type strain Yb2, suggesting that the biotin biosynthesis was defective. The mutant strain Yb2ΔbioF displayed a decreased growth rate at the exponential phase in tryptic soy broth culture and in BeaverBeads Streptavidin treated tryptic soy broth culture, but recovered when biotin was supplemented. In addition, the mutant strain Yb2ΔbioF showed an enhanced biofilm formation, as well as increased adhesion and invasion capacities to duck embryo fibroblasts. Moreover, the mutant strain Yb2ΔbioF exhibited irregular shapes with budding vegetations and relatively thickened cell walls under scanning and transmission electron microscope observation, as well as a reduced capacity to establish systemic infection in a duck infection model. These results provide the first evidence that the *R*. *anatipestifer* AS87_RS09170 gene is responsible for biotin synthesis, bacterial morphology and virulence.

## Introduction

*Riemerella anatipestifer* is a Gram-negative, non-spore-forming, rod-shaped bacterium that mainly affects commercially-important birds such as ducks and turkeys^1,2^. The bacterium is transmitted by contaminated food, water and air. *R*. *anatipestifer* infection occurs as an acute or chronic septicemia characterized by lesions of the liver, heart and spleen. Infections account for major economic losses to the duck industry globally through poor feed conversion and high mortality^3^. Once infection sets within a duck flock, the bacterium can become endemic with repeated infectious episodes possible making eradication difficult. To date, 21 *R*. *anatipestifer* serotypes have been identified with no significant cross-protection reported. Serotypes 1, 2 and 10 are responsible for most of the major *R*. *anatipestifer* outbreaks in China^4^.

Several virulence factors of *R*. *anatipestifer* have been identified, including VapD^5^, CAMP cohemolysin^6^, outer membrane protein A^7^, putative genes associated with lipopolysaccharide (LPS) synthesis^8–10^ and nicotinamidase^11^. In our previous study, 49 virulence-associated genes were identified by transponson mutagenesis. Transponson *Tn*4351 disrupted the AS87_ RS09170 gene, which encodes a predicted 8-amino-7-oxononanoate synthase (AONS). AONS catalyzes the decarboxylative condensation of L-alanine and pimeloyl-CoA to form 8 (S)-amino-7-oxononanoate, the first committed step in conserved biotin biosynthesis. Biotin is an essential cofactor for the biotin-dependent enzymes that are involved in important metabolic pathways such as membrane lipid synthesis, replenishment of the tricarboxylic acid cycle and amino acid metabolism^12–14^. Biotin can be synthesized *de novo* in microorganisms, plants, and fungi. Biotin biosynthesis can be divided into two stages: (1) synthesis of the pimelate precursor and, (2) assemblage of the bicyclic rings of biotin. The final four steps are highly conserved amongst microorganisms and plants. Pimeloyl-CoA is converted to biotin by the activities of AONS, 7,8-diaminopelargonic acid aminotransferase, dethiobiotin synthetase and biotin synthase, which are encoded by *bioF*, *bioA*, *bioD*, and *bioB*, respectively (supplementary Figure S1). Biotin is intimately associated with lipid synthesis where the products form key components of the mycobacterial cell membrane that are critical for bacterial survival and pathogenesis^15^.

In the present study, we described a mutant strain of *R*. *anatipestifer* Yb2ΔbioF in which the homologous *bioF* gene AS87_RS09170 was disrupted. The bacterial growth, protein biotinylation, biofilm formation, adherence and invasion capabilities, morphology, gene expression as well as colonization and development during infection of the mutant strain Yb2ΔbioF were characterized.

## Results

### The AS87_RS09170 gene is highly conserved in *R*. *anatipestifer* strains

**Figure 1:**
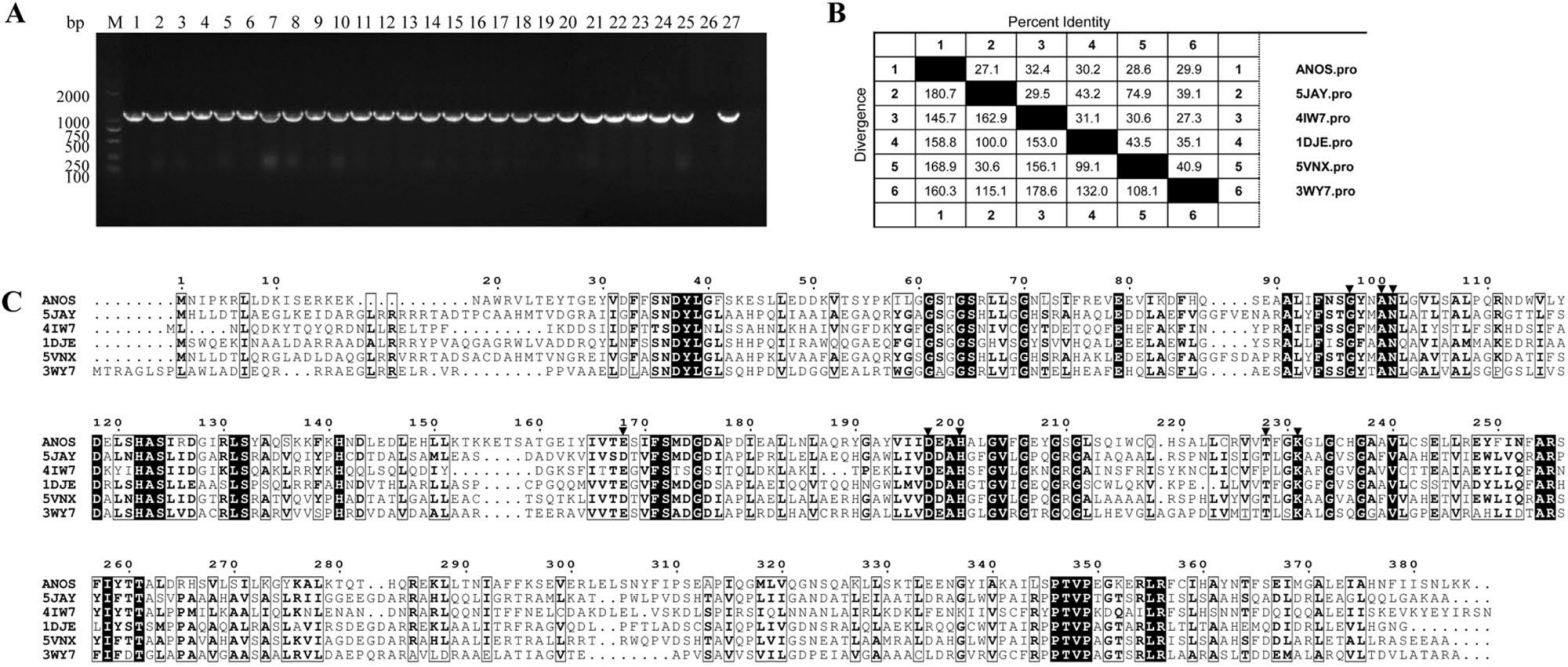
Analyses of *Riemerella anatipestifer* AS87_RS09170 gene and deduced amino acid sequence. (**A**) Polymerase chain reaction (PCR) analysis. The AS87_RS09170 gene was amplified from all *R*. *anatipestifer* strains tested. Lane M, DL2000 DNA Marker (Takara); lanes 1–5: *R*. *anatipestifer* serotype 1 strains GTB1, GTB2, CQ1, CQ3 and NJ4; lanes 6–10, *R*. *anatipestifer* serotype 2 strains GDO-3, JY1, JY2, NJ3 and SC2; lanes 11–15, *R*. *anatipestifer* serotype 10 strains GDO-1, HXb2, YXb1, YXb11 and YXL1; lanes 16–20, *R*. *anatipestifer* serotype 15 strains SQ007, SQ003, SQ004, YGT002 and MA001; lanes 21–25, *R*. *anatipestifer* undefined serotype strains GDO-6, GDO-7, G46, G77 and JY-6; lane 26, negative control of Yb2ΔbioF; lane 27, positive control of Yb2. A 1158-bp fragment was amplified from all 25 *R*. *anatipestifer* strains tested. The full-length gel is presented in Supplementary Figure S2. (**B**) Identity and divergence analysis. The predicted *R*. *anatipestifer* 8-amino-7-oxononanoate synthase (AONS) and other known AONS were compared. The crystallized AONS sequences [*Paraburkholderia xenovorans* (pdb5JAY), *Francisella tularensis* (pdb4IW7), *Escherichia coli* (pdb1DJE), *Burkholderia multivorans* (pdb5VNX) and *Mycobacterium smegmatis* (pdb3WY7)] were retrieved from the Uniprot database and later aligned with Clustal W algorithm in the MegAlign program from the DNASTAR Lasergene suite. (**C**) Multiple sequence alignment. Strictly conserved residues in all sequences are boxed in black. Residues forming the conserved pyridoxal 5′-phosphate binding pocket are indicated by a black inverted triangle (G97, Y98, N101, E168, D197, H200, T228 and K231).

The AS87_RS09170 gene was successfully amplified from all 25 *R*. *anatipestifer* strains tested. A similarity search of the nucleotide databases at the National Center for Biotechnology Information using the Basic Local Alignment Search Tool (BLAST) program showed that the AS87_RS09170 gene exhibited 100% identity with *R*. *anatipestifer* strain NCTC11014 (GenBank accession no. LT906475.1), RA-CH-2 (GenBank accession no. CP004020.1) and ATCC11845 (GenBank accession no. CP003388.1), 99% identity with *R*. *anatipestifer* strain 153 (GenBank accession no. CP007504.1), *R*. *anatipestifer* strain 17 (GenBank accession no. CP007503.1) and RA-GD (GenBank accession no. CP002562.1), 94% identity with *R*. *anatipestifer* strain CH3 (GenBank accession no. CP006649.1), RA-CH-1 (GenBank accession no. CP003787.1) and HXb2 (GenBank accession no. CP011859.1). These data indicated that the AS87_RS09170 gene is highly conserved in *R*. *anatipestifer* strains. The gene encodes a predicted 385-amino acid AONS, shares 27.1% to 32.4% sequence identity with AONS from other microorganisms, from which the protein has been crystallized, including *Paraburkholderia xenovorans* (PDB accession no. 5JAY [27.1% identity]), *Francisella tularensis* (PDB accession no. 4IW7 [32.4% identity]), *Escherichia coli* (PDB accession no. 1DJE [30.2% identity]), *Burkholderia multivorans* (PDB accession no.5VNX [28.6% identity]) and *Mycobacterium smegmatis* (PDB accession no. 3WY7 [29.9% identity]). Moreover, the sequence alignment demonstrated that the putative *R*. *anatipestifer* AONS protein harbored the conserved pyridoxal 5′-phosphate binding pocket composed of amino acids G97, Y98, N101, E168, D197, H200, T228 and K231 (Fig. 1).

### Characterization of the mutant strain Yb2ΔbioF

**Figure 2:**
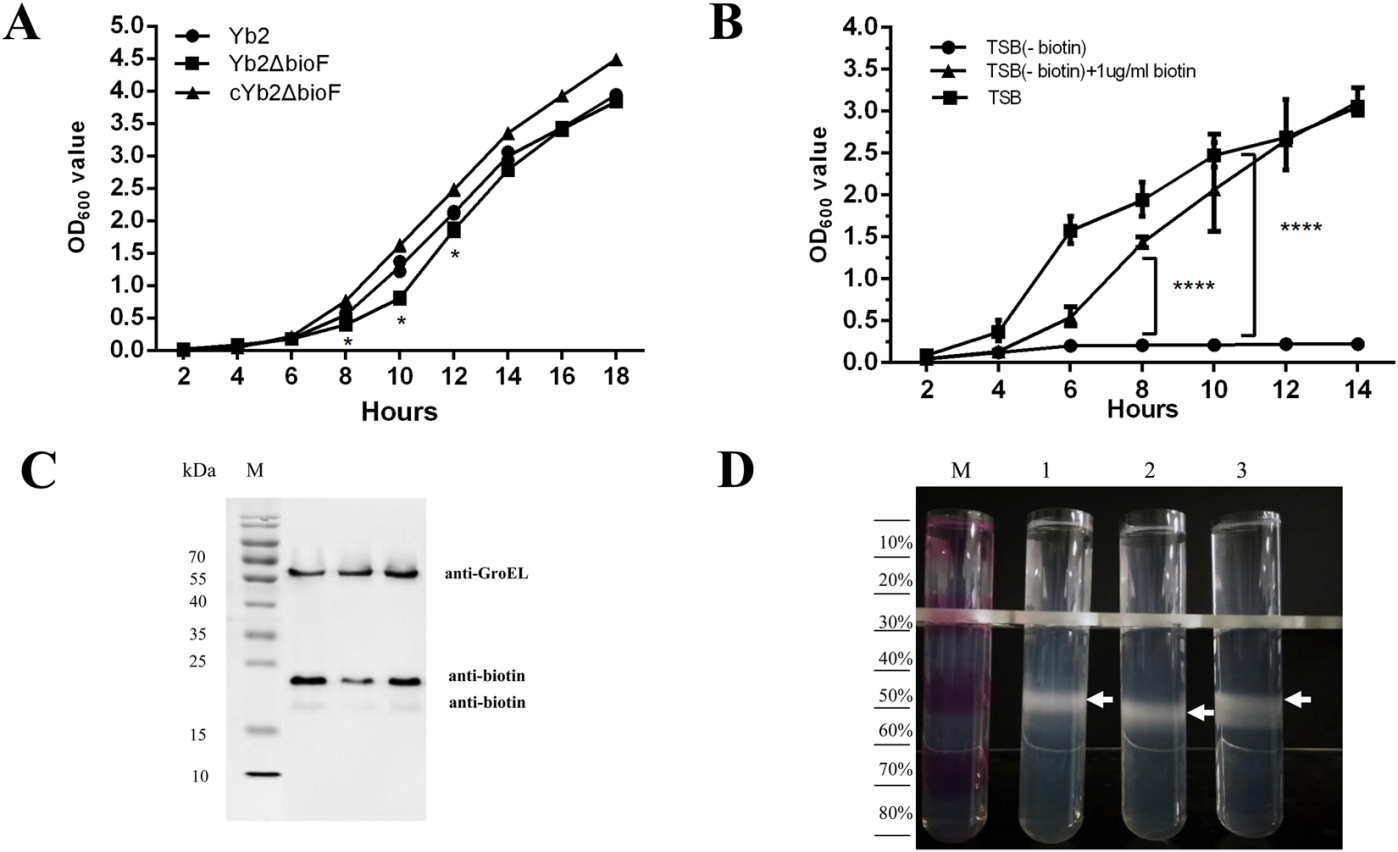
Characterization of mutant strain Yb2ΔbioF. (**A**) Growth curves of Yb2, Yb2ΔbioF and cYb2ΔbioF in tryptic soy broth (TSB). (**B**) Growth curves of Yb2ΔbioF in TSB, TSB-biotin and TSB-biotin+biotin. Each point represents the mean ± standard for triplicate samples. (*p < 0.05; ***p < 0.001). (**C**) Immunoblot analysis of the biotinylated protein. Whole-cell proteins of each strain were separated on sodium dodecyl sulfate polyacrylamide gel electrophoresis (SDS-PAGE) gel, transferred to nitrocellulose membrane, blotted with streptavidin peroxidase, and visualized by chemiluminescence. *Riemerella anatipestifer* GroEL was used as a protein loading control. The full-length blots are presented in Supplementary Figure S3. (**D**) Percoll density gradient centrifugation of the *R. anatipestifer* strains. The gradient is visualized using fuchsine-stained layers in the marker (M). Lane 1, the wild-type strain Yb2; lane 2, the mutant strain Yb2ΔbioF; lane 3, the complemented strain cYb2ΔbioF.

The mutant strain Yb2ΔbioF was viable when grown in tryptic soy broth (TSB) medium, and reached a similar stationary phase in TSB compared with the wild-type (WT) strain Yb2. However, the growth rate of the mutant was significantly reduced in the logarithmic phase between 8 and 12 h. Transformation of the mutant strain Yb2ΔbioF with the AS87_RS09170 gene complemented the growth defect in TSB medium (Fig. 2A). To further access the effect of biotin biosynthesis on *R*. *anatipestifer* growth, we assessed the growth capacity of the mutant strain Yb2ΔbioF in either TSB, the BeaverBeads Streptavidin treated TSB (designated as TSB-biotin) or the BeaverBeads Streptavidin treated TSB replete with biotin (designated as TSB-biotin+biotin). As shown in Fig. 2B, the growth deficiency of the mutant strain Yb2ΔbioF in the biotin depleted TSB was restored when biotin was supplemented at a final concentration of 1.0 μg/ml.

We also assessed the impact of biotin deficiency on protein biotinylation by anti-biotin immunoblotting. As shown in Fig. 2C, a deficiency of immunoreactivity in two bands corresponding with two proteins was detected in mutant strain Yb2ΔbioF, indicating that the quantity of biotinylated protein decreased. The electrophoretic mobility and anti-biotin immunoreactivity of these proteins suggested that they correspond to two isoforms of acetyl-CoA carboxylase, which are predicted to be biotinylated in *R*. *anatipestifer*. Liquid chromatography-electrospray ionization-tandem mass spectrometry (LC-ESI-MS/MS) analysis of the BeaverBeads Streptavidin enriched protein further confirmed the anti-biotin immunoreactive protein was acetyl-CoA carboxylase. The levels of biotinylated acetyl-CoA carboxylase were recovered in the complemented strain cYb2ΔbioF (Fig. 2C).

Percoll density gradient centrifugation analyses showed that the density of Yb2ΔbioF was slightly changed compared to its WT strain Yb2 (Fig. 2D). Yb2 cells settled at the 40–50% interface, whereas the Yb2ΔbioF mutant cells settled at the 60% interface. In addition, the appearance was more diffuse for the WT strain Yb2, compared to the mutant strain Yb2ΔbioF.

### The mutant strain Yb2ΔbioF presented an increased biofilm formation

**Figure 3:**
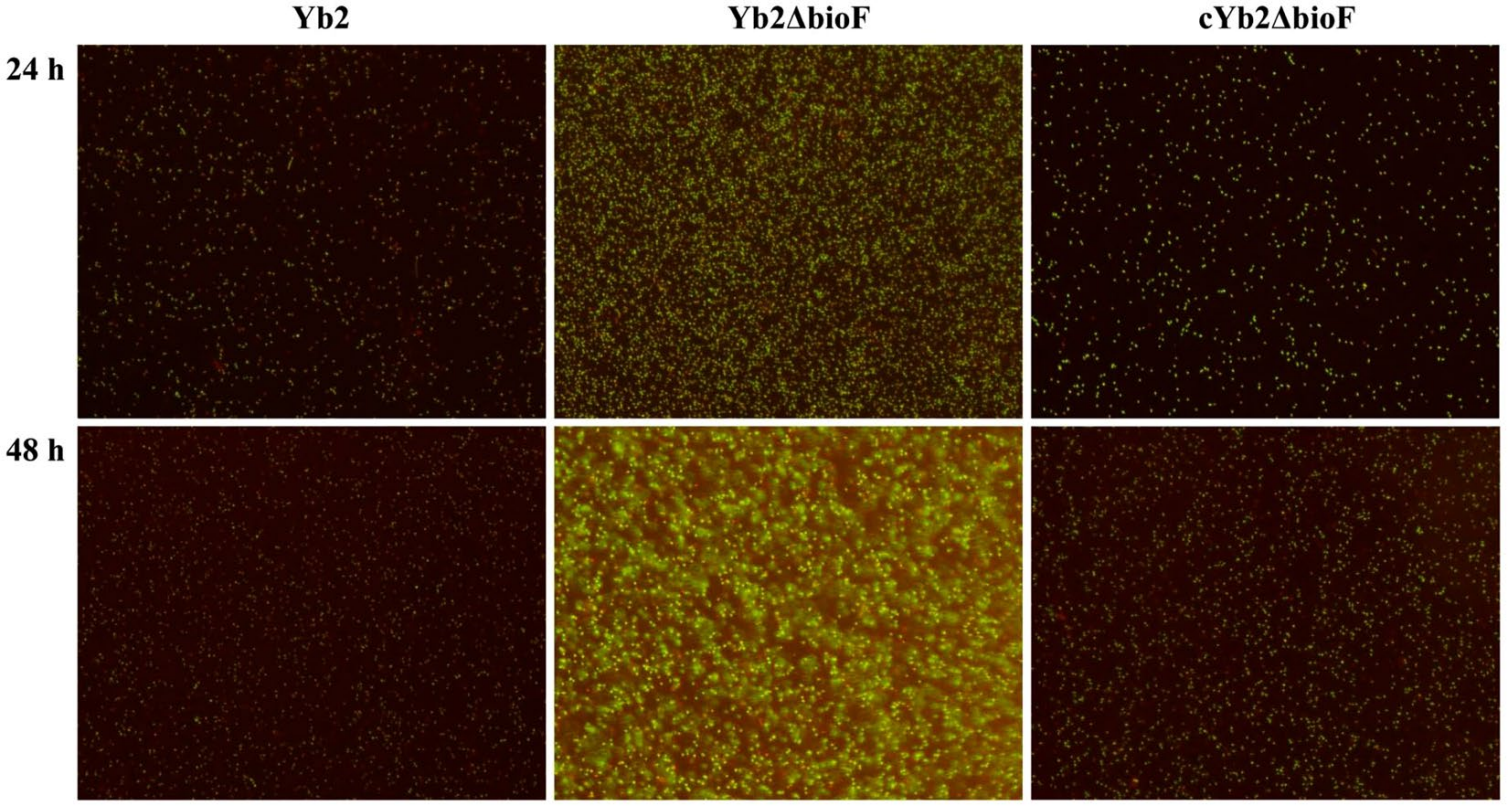
Observation of the biofilm formation. The biofilm formation of the wild-type strain Yb2, mutant strain Yb2ΔbioF and complemented strain cYb2ΔbioF was determined using an Live/dead BacLight Bacterial Viability staining and observed under fluorescence microscope at a 200× magnification, which revealed that the mutant strain Yb2ΔbioF had a stronger ability to form biofilm than the wild-type strain Yb2.

The ability of the mutant strain Yb2ΔbioF to form a biofilm on borosilicate glass surface was examined by fluorescence microscopy. As shown in Fig. 3 with Live/dead BacLight Bacterial Viability staining, the mutant strain Yb2ΔbioF presented a significantly increased abundance of live cells, as compared with the WT strain Yb2 and complemented strain cYb2ΔbioF at 24 h incubation. At 48 h incubation, the mutant strain Yb2ΔbioF produced a mature biofilm, which was structured with numerous microcolonies encased in a thick opaque extracellular matrix, whereas the WT and complemented strain cYb2ΔbioF failed to form a layer of singly attached biofilm on the surface of borosilicate glass.

### Inactivation of the AS87_RS09170 gene increased the bacterial adherence and invasion abilities

**Figure 4:**
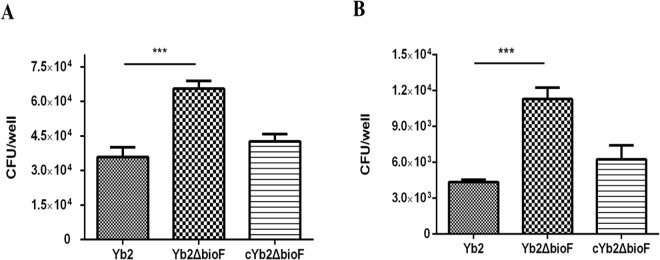
Bacterial adherence and invasion assays. (**A**) Adherence assay. (**B**) Invasion assay. Strains Yb2, Yb2ΔbioF and cYb2ΔbioF were tested on duck embryo fibroblast cells. The data represent the number of bacteria bound to or invaded into duck embryo fibroblast cells in each well of a 24-well plate. The error bars represent standard deviation calculated from three independent experiments performed in triplicate (***p < 0.001).

To determine whether disruption of the AS87_RS09170 gene affected bacterial adherence and invasion capacities, the WT strain Yb2, mutant strain Yb2ΔbioF and complemented strain cYb2ΔbioF were tested on duck embryo fibroblast cells. When infected at 100 MOI, the host cell-associated Yb2ΔbioF bacteria were counted as 65,600 ± 8,015 colony forming units (CFU)/well, which was significantly increased in comparison with that of the WT strain Yb2 (35,933 ± 10,277 CFU/well). After an additional 1 h of incubation with gentamicin, the invaded Yb2ΔbioF bacteria were counted as 11,293 ± 2,367 CFU/well, approximately 3-fold higher than that of the WT strain Yb2 (4,360 ± 457 CFU/well). The results showed that adherence and invasion capacities of Yb2ΔbioF were significantly increased, compared with those of the WT strain. The complemented strain cYb2ΔbioF exhibited WT levels of adherence and invasion (Fig. 4).

### Morphology and ultrastructure observation

**Figure 5:**
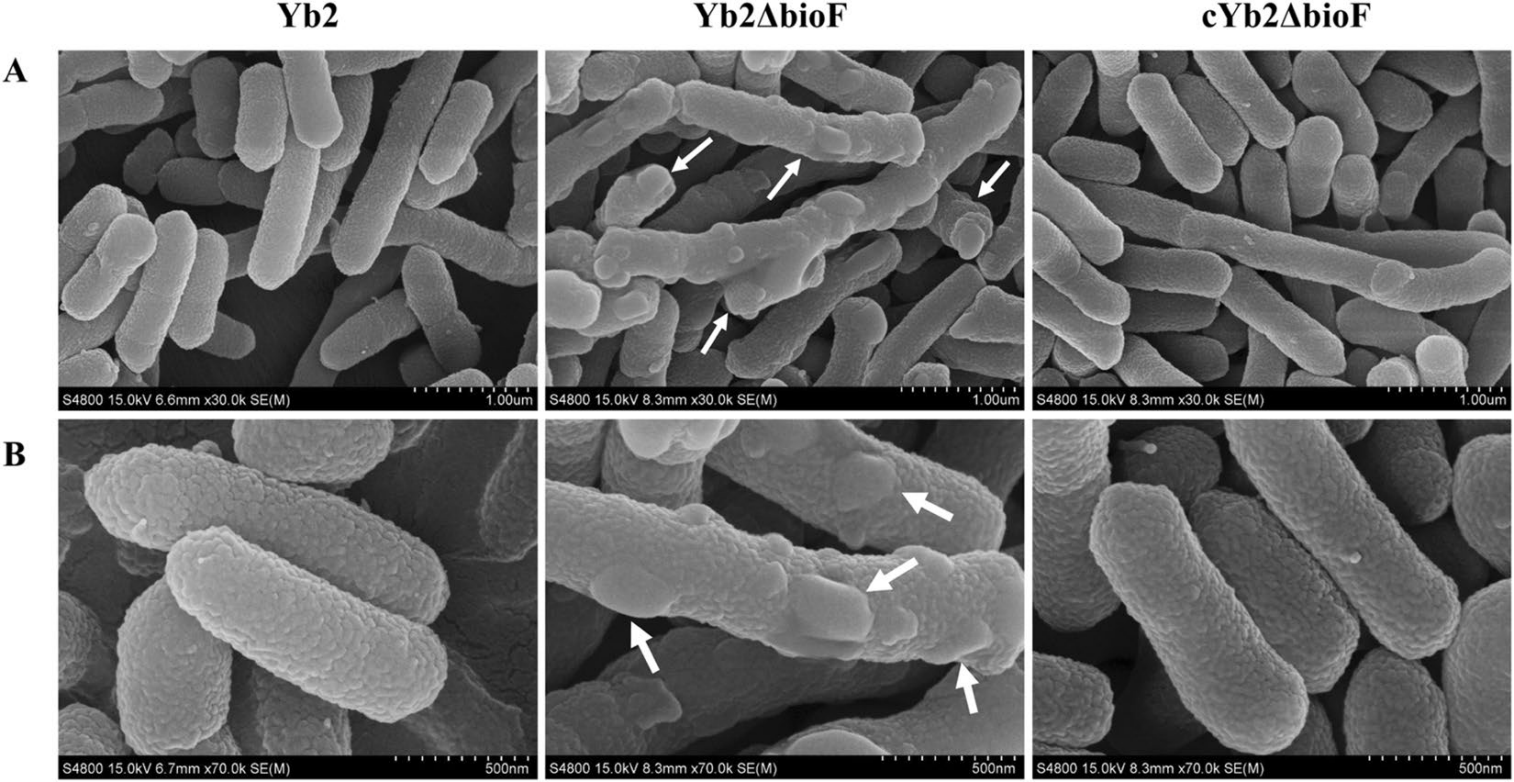
Morphological observation. (**A**) Low magnification; (**B**) high magnification. The shapes and sizes of wild-type strain Yb2, mutant strain Yb2ΔbioF and complemented strain cYb2ΔbioF are shown. The images were captured using a scanning electron microscope. Budding vegetations (white arrows) on the surface of mutant strain Yb2ΔbioF were observed.

The morphology of the *R*. *anatipestifer* cells was observed under scanning microscopy. As shown in Fig. 5, WT cells were regular rod-shaped with round ends, consistent with a previous report^16^. The mutant strain Yb2ΔbioF cells remained bacilliform, but with irregular shapes and multiple budding vegetations. There were no obvious perforations in the cellular surface. The complemented strain cYb2ΔbioF recovered the morphology and surface features.

**Figure 6:**
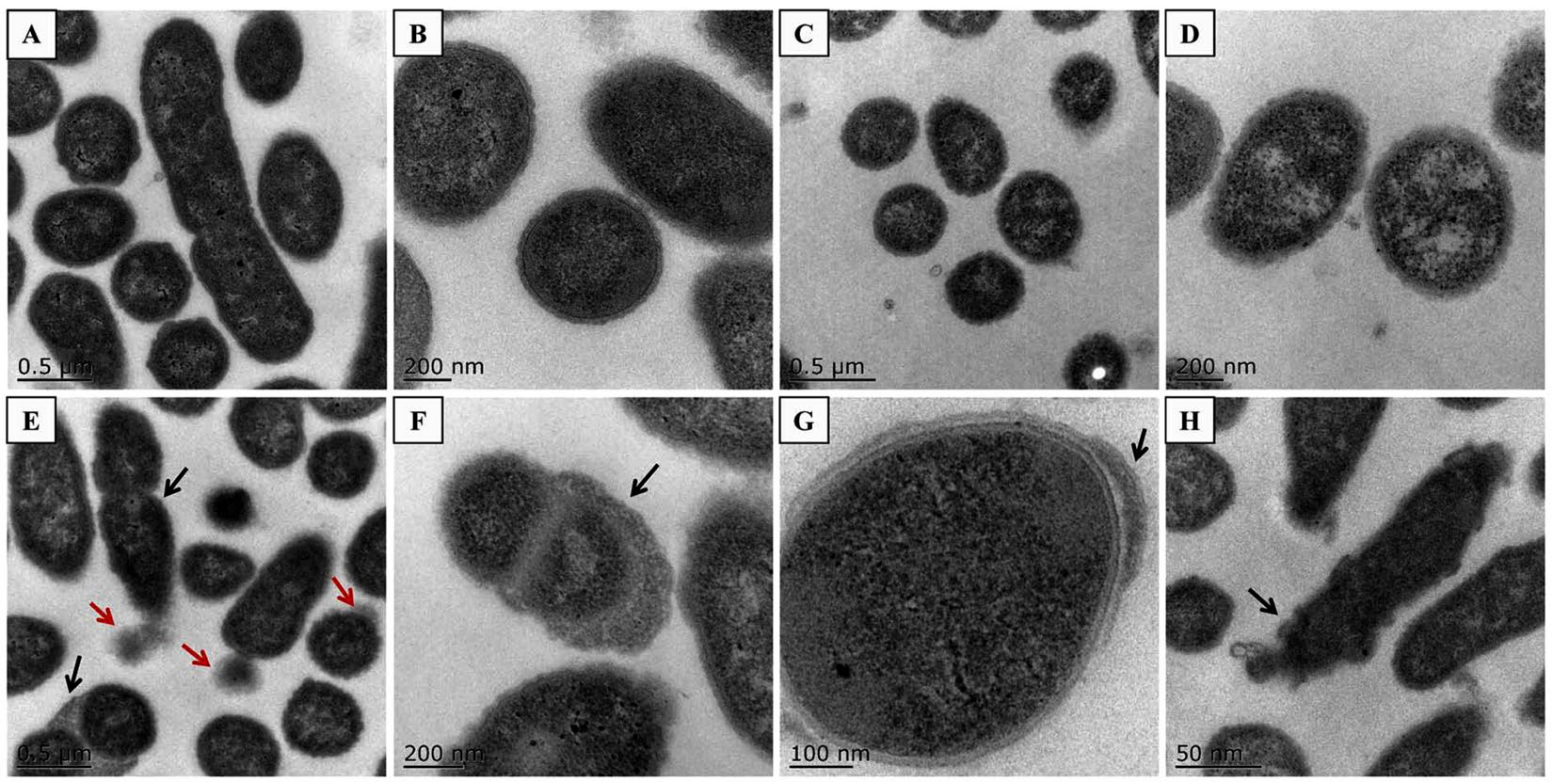
Cellular ultrastructure observation. (**A**,**B**) Phenotypes of wild-type strain Yb2. (**A**) Integral and regularly shaped cells, a few cells were in division; (**B**) regularly shaped cells at higher magnification. (**C**,**D**) Phenotypes of complemented strain cYb2ΔbioF. (**C**) Integral and regularly shaped cells; (**D**) regularly shaped cells at higher magnification. (**E**–**H**) Phenotypes of mutant strain Yb2ΔbioF. (**E**) Dropped vegetations (red arrows) and most cells were in division (black arrows); (**F**) relatively thickened cell walls (black arrow); (**G**) partially thickened cell walls (black arrow); (**H**) irregular shapes (black arrow).

The cellular ultrastructure was observed under a transmission electron microscope (Fig. 6). WT Yb2 cells were integral with clear structures, and few were undergoing division. The mutant strain Yb2ΔbioF cells presented as irregular shapes with budding vegetations, and relatively thickened cell walls. The numbers of cells undergoing division were increased compared with the WT strain Yb2.

### Extraction and identification of membrane proteins

**Figure 7:**
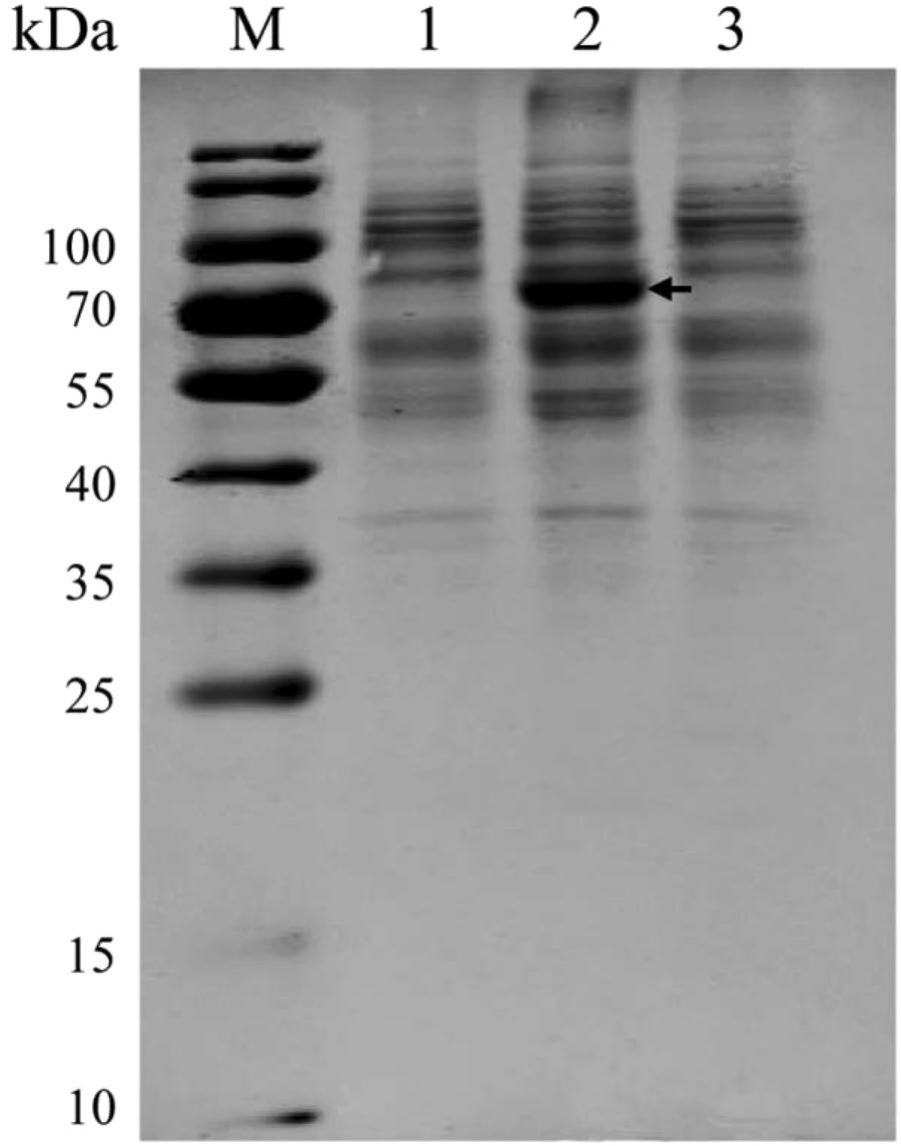
Analysis of membrane proteins. Lane M, weight marker, lane 1, samples from the wild-type strain Yb2, lane 2, samples from the mutant strain Yb2ΔbioF, lane 3, samples from the complemented strain cYb2ΔbioF. The arrow indicates the band appeared only in the mutant strain Yb2ΔbioF. The full-length gel is presented in Supplementary Figure S4.

Membrane proteins were extracted from the WT strain Yb2, mutant strain Yb2ΔbioF and complemented strain cYb2ΔbioF, followed by sodium dodecyl sulfate polyacrylamide gel electrophoresis (SDS-PAGE) analysis. As shown in Fig. 7, a 70-kDa band was significantly displayed in the purified membrane proteins from mutant strain Yb2ΔbioF, but was absent in both the WT strain Yb2 and complemented strain cYb2ΔbioF. The band corresponding to 70-kDa was excised from gel and analyzed by LC-ESI-MS/MS, and identified proteins are summarized in supplementary Table S2. Among the 133 proteins analysed, 93 (70%), 12 (9%), 11 (8%), four (3%) and 13 (10%) were predicted to be cytoplasmic, cytoplasmic membrane, outer membrane, periplasmic, and of unknown locations, respectively.

### Determination of the differentially expressed genes

Strand-specific Illumina RNA-Seq analysis was used to investigate the differentially expressed genes between the WT strain Yb2 and mutant strain Yb2ΔbioF. In total, 13 and 26 genes were up-regulated and down-regulated in the mutant, respectively, in comparison to the WT strain. Quantitative polymerase chain reaction (qPCR) further confirmed that transcription of ten genes in Yb2ΔbioF were down-regulated by over 2-fold (Table 1). Of these, the proteins encoded by AS87_RS01370, AS87_RS01365, AS87_RS01360, AS87_RS01355 and AS87_RS01350 were annotated as vault protein inter-alpha-trypsin (VIT) family protein, sulfite exporter TauE/SafE family protein, efflux efflux resistance-nodulation-division (RND) transporter periplasmic adaptor subunit, TolC family protein and CusA/CzcA family heavy metal efflux RND transporter, respectively, indicating that the AS87_RS09170 gene disruption affected expression of genes primarily responsible for transmembrane transport.

**Table 1:** Real-time PCR verification of differentially expressed genes in mutant strain Yb2ΔbioF.

Gene locus^a^	Description of genes	2^−ΔΔCt b^
AS87_RS05800	hypothetical protein	1.69
AS87_RS04150	glycoside hydrolase	1.55
AS87_RS04120	exosortase F system-associated protein	1.53
AS87_RS09460	hypothetical protein	1.51
AS87_RS09445	hypothetical protein	1.34
AS87_RS03745	NADH-quinone oxidoreductase subunit A	1.29
AS87_RS05725	hypothetical protein	1.27
AS87_RS05655	haloacid dehalogenase	1.16
AS87_RS02380	ABC transporter ATP-binding protein	1.14
AS87_RS06190	ABC transporter ATP-binding protein	1.13
AS87_RS03960	type B 50S ribosomal protein L31	0.99
AS87_RS01395	transcription activator effector-binding protein	0.97
AS87_RS10270	hypothetical protein	0.96
AS87_RS02825	HIT family protein	0.96
AS87_RS07030	mannose-6-phosphate isomerase	0.95
AS87_RS10115	hypothetical protein	0.91
AS87_RS01890	hypothetical protein	0.86
AS87_RS10350	IS982 family transposase	0.86
AS87_RS06890	hypothetical protein	0.85
AS87_RS02260	polyphosphate kinase 2	0.84
AS87_RS01190	DUF465 domain-containing protein	0.79
AS87_RS00965	50S ribosomal protein L19	0.75
AS87_RS05265	hypothetical protein	0.67
AS87_RS09430	hypothetical protein	0.61
AS87_RS07650	30S ribosomal protein S20	0.60
AS87_RS00160	monofunctional biosynthetic peptidoglycan transglycosylase	0.54
AS87_RS08680	TonB-dependent receptor	0.51
AS87_RS02480	hypothetical protein	0.50
AS87_RS09175	dethiobiotin synthase	0.50
AS87_RS01370	VIT family protein	0.48
AS87_RS07305	cytochrome c nitrite reductase small subunit	0.42
AS87_RS01365	sulfite exporter TauE/SafE family protein	0.42
AS87_RS01360	efflux RND transporter periplasmic adaptor subunit	0.35
AS87_RS01355	TolC family protein	0.35
AS87_RS01350	CusA/CzcA family heavy metal efflux RND transporter	0.34
AS87_RS10025	hypothetical protein	0.31
AS87_RS07820	alpha/beta hydrolase	0.27
AS87_RS09180	adenosylmethionine–8-amino-7-oxononanoate aminotransferase BioA	0.20
AS87_RS09185	hypothetical protein	0.15

^a^Based on *Riemerella anatipestifer* Yb2 genome (accession number: CP007204).

^b^Results are presented as 2^ΔΔCt^. Figure = 1 indicated that the gene is expressed similarly in both mutant strain Yb2ΔbioF and wild-type strain Yb2, figures >1 indicated that the gene is over expressed in mutant strain Yb2ΔbioF, and figures <1 indicated that the gene is expressed less in mutant strain Yb2ΔbioF.

### Determination of the bacterial virulence

**Figure 8:**
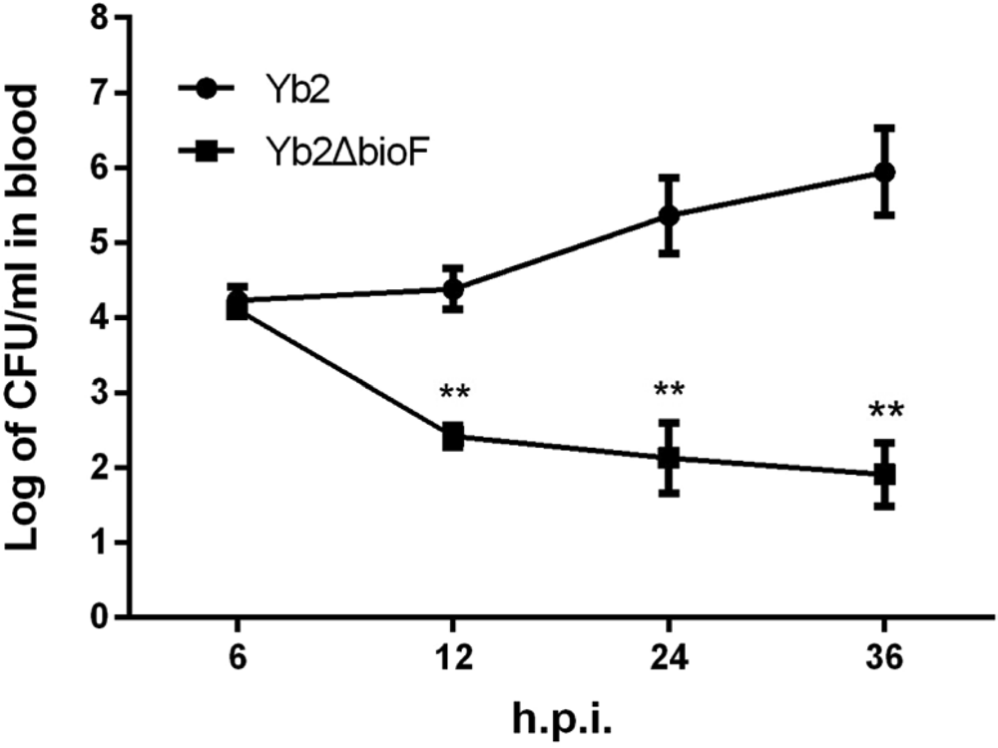
Bacterial loads in blood of *Riemerella anatipestifer* infected ducks. Six ducks each were infected with the bacterial culture at a dose of 1 × 10^8^ CFU. The bacterial loads in blood samples were counted at 6 hpi, 12 hpi, 24 hpi, and 36 hpi. Data represent mean ± standard deviation, and were analyzed using two-tailed independent Student’s *t* test (**p < 0.01).

Our previous study revealed that disruption of the AS87_RS09170 gene resulted in a 768,000-fold attenuation of virulence, compared with the WT strain Yb2^17^. To further investigate the role of the AS87_RS09170 gene in systemic invasion and dissemination, the bacterial loading in blood of infected ducks was quantified. Results showed that bacterial recovery of the WT strain Yb2 maintained an increase of up to 1.82 × 10^6^ CFU/ml at 36 hpi. In contrast, the recovery of the mutant strain Yb2ΔbioF was gradually decreased post infection, and was measured as 270 CFU/ml, 213 CFU/ml, and 124 CFU/ml at 12, 24, and 36 hpi, respectively. The mutant strain was significantly attenuated (Fig. 8).

## Discussion

Biotin, an indispensable nutrient found in all living cells, is synthesized *de novo* in many microorganisms, plants, and a few fungi. The early steps of the pathway are responsible for the synthesis of pimelic acid, whereas the last four steps are responsible for assembly of the rings. *R*. *anatipestifer* possesses all of the putative proteins (BioF, BioA, BioD, BioB) of the late steps of biotin biosynthesis. In contrast, *R*. *anatipestifer* lacks homologous genes of *bioCHIW* required for pimelate synthesis, suggested that pimeloyl-CoA could be formed by the enzymes of fatty acid synthesis, which is consistent with the findings of Steven Lin^18^. Here, we characterized a mutant strain Yb2ΔbioF in which the putative *bioF* gene responsible for biotin biosynthesis was inactivated. We screened a library of random transposon mutants in an animal infection model and obtained an attenuated mutant, Yb2ΔbioF, with the AS87_RS09170 gene disrupted by the Tn4351 insertion in the open reading frame^17^. This mutant showed a decreased growth rate in TSB at the exponential phase (Fig. 2A) and reduced quantities of biotinylated proteins. The growth of the mutant strain Yb2ΔbioF in biotin-depleted TSB was almost halted, but recovered with addition of biotin (Fig. 2B). Subsequently, we showed that the biotin deficiency resulted in an increase in biofilm formation (Fig. 3), as well as increased capacity for adhesion and invasion to duck embryo fibroblast (Fig. 4). Our data showed that biotin deficiency also led to changes of cell morphology (Fig. 5), resulting in irregular shapes with budding vegetations and relatively thickened cell walls (Fig. 6). Genetic complementation with the AS87_RS09170 gene restored the growth rate, protein biotinylation, biofilm formation and adhesion/invasion capacities, as well as cell morphology, confirming that deficient biotin synthesis is responsible for the observed phenotypes.

Biotin acts as a carbon dioxide carrier during carboxylation, which comprises decarboxylation and transcarboxylation reactions responsible for biosynthesis of fatty acids, gluconeogenesis and amino acid metabolism^19^. Based on the genomic annotation analysis, *R*. *anatipestifer* genome has two putative biotinylated proteins of acetyl-CoA carboxylase biotin carboxyl carrier protein (AS87_RS06990) and acetyl-CoA carboxylase biotin carboxylase subunit (AS87_RS06995). Our results confirmed that two biotinylated bands at 18 kDa and 24 kDa, which correspond to the sizes of above two proteins, were clearly reduced in the mutant strain Yb2ΔbioF in comparison with the WT strain Yb2 (Fig. 2C). LC-ESI-MS/MS further confirmed that the 24 kDa protein was acetyl-CoA carboxylase, but the 18 kDa protein was not determined due to its lower abundance, suggesting that the AS87_RS09170 gene product participates in biotin biosynthesis and protein biotinylation. Alterations in biotinylated protein levels caused by biotin biosynthesis deficiency were also observed in *Mycobacterium tuberculosis* and *Arabidopsis thaliana*^13,20^.

Cell surface adhesion and cell aggregation initiate bacterial biofilm formation^21^. Our results demonstrated that AS87_RS09170 disruption significantly increased the formation of solid biofilms, suggesting that the cell-cell and cell surface interactions of the mutant strain Yb2ΔbioF were increased. The fact that absence of the AS87_RS09170 protein induced rather than repressed aggregation indicates that AS87_RS09170 protein itself is not directly involved in cell-cell adhesion. In addition, the hydrophobic experiment revealed that disruption of the AS87_RS09170 gene did not affect bacterial hydrophobicity (data not shown), indicating that it is unlikely that the biofilm formation was mediated by bacterial cell surface hydrophobic properties. Moreover, the capacities of mutant strain Yb2ΔbioF to adhere to and invade duck embryo fibroblast cells were sharply increased compared those of the WT strain Yb2, which is consistent with the observation that the mutant strain increased the liquid-solid interface biofilm formation. Further investigation revealed that the dramatic change of the *R*. *anatipestifer* cell surface morphology from regular to irregular with budding vegetations was caused by AS87_RS09170 inactivation. Therefore, it is most likely that the AS87_RS09170 protein modulated bacterial biofilm formation and adherence/invasion capacities by affecting the cell surface morphology.

In addition to acting as a coenzyme for carboxylases, biotin also plays a unique role in regulation of genes expression^22^. qPCR analysis revealed that deleting the AS87_RS09170 gene down-regulated ten genes, five of which encoded efflux RND transporter-associated proteins. The differential expression of these genes may critically change bacterial cell surface properties. Further membrane protein analysis revealed the presence of many cytoplasmic proteins in the mutant strain Yb2ΔbioF, suggesting they may have leaked from cytoplasm, and the differentially expressed genes associated with cellular transport played a role in this leakage. Therefore, the cytoplasmic protein leakage in mutant strain Yb2ΔbioF might result in budding vegetations on the bacterial surface. Considered together, these results suggest that the AS87_RS09170 gene is responsible for the biotin synthesis and gene expression, which caused the defects in bacterial cell surface structures, led to leakage of cytoplasmic proteins and consequent increased biofilm formation, bacterial adherence and invasion.

The manifestation of *R*. *anatipestifer* infection is characterized as septicemia, and virulence and pathogenesis of this species are tightly associated with the ability of the bacteria to colonize and develop in the host. We found that the mutant strain Yb2ΔbioF failed to colonize and multiply in ducks, evident by decreased bacterial loadings in the blood of Yb2ΔbioF-infected birds. In addition, the mutant strain Yb2ΔbioF exhibited 768,000-fold attenuated virulence. Our results indicated that the biotin synthesis plays a key role in the survival of *R*. *anatipestifer* during the infection. A previous study revealed that biotin biosynthesis of *Mycobacterium marinum* plays an important role in colonization and development of the host^23^.

In summary, the present study demonstrated that the *R*. *anatipestifer* AS87_RS09170 gene is responsible for biotin synthesis, which plays important roles in bacterial growth, protein biotinylation and establishment of systemic infection in ducks. Furthermore, our finding provided the first evidence demonstrating that biotin biosynthesis is essential for bacterial surface morphology in *R*. *anatipestifer*.

## Materials and Methods

### Ethical considerations

Duck eggs were obtained from the Zhuang Hang Duck Farm (Shanghai, China) and hatched in an incubator (Deguang, Shandong, China) in our laboratory. One-day old Cherry Valley ducks were obtained from the Zhuang Hang Duck Farm (Shanghai, China) and raised under controlled temperature (28–30 °C). The ducks were housed in cages with free access to food and water under the conditions of biological safety. Animal experiments were conducted according to the Institutional Animal Care and Use Committee (IACUC) guidelines set by the Shanghai Veterinary Research Institute, Chinese Academy of Agricultural Sciences (CAAS). The animal study protocol used in the present study was approved by the IACUC of the Shanghai Veterinary Research Institute, CAAS, China (permit no. Shvri-po-0176). All surgeries were performed under sodium pentobarbital anesthesia, and all efforts were made to minimize suffering.

### Bacterial strains, plasmids and culture conditions

The bacterial strains, plasmids and primers used in the present study are listed in Table 2. *R*. *anatipestifer* Yb2 is the serotype 2 WT virulent strain, and the mutant strain RA1893 (Yb2ΔbioF in the present study) was derived from this strain by *Tn4351* transposon insertion^17^. *R*. *anatipestifer* strains were grown on tryptic soy agar (TSA, Difco, NJ, USA) at 37 °C for 24 h in 5% CO_2_ or in tryptic soy broth (TSB, Difco) at 37 °C with shaking at 200 rpm for 8 to 12 h. To remove the biotin from TSB, 50 ml TSB was mixed with 300 μl of BeaverBeads Streptavidin (BeaverBio, Suzhou, China) and incubated with shaking at 4 °C for 12 h. After the beads were removed, the TSB medium was passed through 0.22 μm filter, subsequently designated as TSB-biotin. *Escherichia coli* strains were grown at 37 °C on Luria-Bertani (LB) plates or in LB broth. Antibiotics were added at the appropriate concentrations when required: ampicillin (100 μg/ml), erythromycin (0.5 μg/ml), kanamycin (50 μg/ml) and cefoxitin (5 μg/ml).

**Table 2:** Strains, plasmids and primers used in this study.

Strains, plasmids or primers	Characteristics	Sources or references
**Strains**		
Yb2	*Riemerella anatipestifer* serotype 2 strain	^39^
Yb2ΔbioF	Tn*4351* insertion mutant of *R*. *anatipestifer*Yb2, *bioF*::Tn	^17^
cYb2ΔbioF	Mutant strain RA1893 carrying plasmid pCP29-*bioF*	This study
*Escherichia coli* S17-1	λ*pirhsdRprothi*; chromosomally integrated RP4-2, Tc::Mu Km::Tn7	^7^
**Plasmids**		
pCP29	ColE1 ori (pCP1 ori); Ap^r^(Em^r^); *E*. *coli*-*F*. *johnsoniae* shuttle plasmid	^7^
pCP29-*bioF*	pCP29 containing *ompA* promoter and *bioF* ORF, cfxA^r^(Ap^r^)	This study
**Primers**		
*ompA* promoter P1	5′-CAGGTACCATAGCTAAAATTTTGGCAGTAAC-3′ (KpnΙ site underlined)	^7^
*ompA* promoter P2	5′-CGACTCGAGCATTCCAATTCTCTTATTATC-3′ (XhoΙ site underlined)	^7^
16S rRNA F	5′-GAGCGGTAGAGTATCTTCGGATACT-3′	^8^
16S rRNA R	5′-AATTCCTTTGAGTTTCAACCTTGCG-3′	^8^
*bioF* comp-F	5′-CCGCTCGAGATGAATATTCCCAAAAGATTGTTAGAT-3′ (XhoΙ site underlined)	This study
*bioF* comp-R	5′-CATGCATGCTTATTTCTTTAAATTAGAAATGATAAAATTGTGGGC-3′ (SphΙ site underlined)	This study

The shuttle plasmid pCP29 was used for complementation of mutant strain Yb2ΔbioF^24^. The AS87_RS09170 open reading frame was amplified from the WT strain Yb2 using primers bioF comp-F/bioF comp-R. The PCR product was inserted into pCP29 at XhoI and SphI restriction sites, producing recombinant plasmid pCP29-*bioF*. Expression of the AS87_RS09170 gene was under the control of the *ompA* promoter, as described previously^25^. Plasmids were first introduced into *E*. *coli* S17-1 by transformation. Next, they were transferred into mutant strain Yb2ΔbioF by conjugation. Transformants were selected on TSA containing 5 μg/ml cefoxitin and 50 μg/ml kanamycin, and identified by PCR amplification using primers bioF comp-F/bioF comp-R and RA 16S rRNA-F/RA 16S rRNA-R. The complemented strain is subsequently designated as cYb2ΔbioF.

### Analyses of AS87_RS09170 gene distribution in *R*. *anatipestifer* strains and deduced amino acid sequence

Genomic DNA of 25 *R*. *anatipestifer* strains with different serotypes was isolated using the TIANamp Bacteria DNA kit (Tiangen, Beijing, China) according to the manufacturer’s instruction. The AS87_RS09170 gene in *R*. *anatipestifer* strains was amplified using primers bioF comp-F and bioF comp-R, followed by agarose gel electrophoresis. The crystallized AONS sequences [*Paraburkholderia xenovorans* (pdb5JAY), *Francisella tularensis* (pdb4IW7), *E*. *coli* (pdb1DJE), *Burkholderia multivorans* (pdb5VNX), and *Mycobacterium smegmatis* (pdb3WY7)] were retrieved from Universal Protein Resource (UniProt) (http://www.uniprot.org/). Similarity of the predicted *R*. *anatipestifer* AONS sequence and other crystallized AONS sequences was analyzed using the Clustal W algorithm in the MegAlign program from the DNASTAR Lasergene suite, and rendered with Espript^26^.

### Bacterial growth curves

The WT strain Yb2, mutant strain Yb2ΔbioF and complemented strain cYb2ΔbioF were cultured in TSB at 37 °C with shaking, and the bacterial growth was measured at an optical density at 600 nm (OD_600_) as described previously^27^. Equal amounts of each bacterial culture were then transferred into fresh TSB medium at a ratio of 1:100 (v/v) and incubated at 37 °C, with shaking at 200 rpm. The OD_600_ value was measured at 2 h intervals for 16 h using a spectrophotometer (BIO-RAD, USA). To further evaluate the effect of biotin on bacterial growth, the growth of the mutant strain Yb2ΔbioF in TSB, TSB-biotin and TSB-biotin with addition of biotin at final concentrations of 1.0 μg/ml were measured as described above.

### Characterization of biotinylated protein

To detect the biotinylated proteins in the bacteria, the whole-cell proteins of the WT strain Yb2, mutant strain Yb2ΔbioF and complemented strain cYb2ΔbioF were extracted using a bacterial total proteins extraction kit (BestBio, Shanghai, China), subjected to SDS-PAGE and transferred into a nitrocellulose membrane (Whatman, Sigma-Aldrich). The membrane was blocked for 1 h at room temperature in phosphate-buffered saline (PBS, pH7.2) containing 1% bovine serum albumin (BSA), rinsed with PBST (PBS containing 0.05% Tween 20), and then incubated with Ultrasensitive Streptavidin-Peroxidase polymer (Sigma-Aldrich) diluted 1:1000 in PBS with 0.05% Tween 20 at room temperature for 1 h. After washing six times with PBST, the membrane was incubated with a basic ECL subsolution kit (share-bio, Shanghai, China) and blots were visualized using the Chemiluminescent Imaging System (Tanon, Shanghai, China). The mouse anti-GroEL monoclonal antibody (prepared in our laboratory previously^28^) and a horseradish peroxidase-conjugated goat anti-mouse IgG polyclonal antibody (KPL, Milford, MA, USA) were used to probe GroEL, a protein loading control. To identify the biotinylated protein, the whole-cell proteins of the Yb2 were incubated with the BeaverBeads Streptavidin (BeaverBio) with shaking at 4 °C for 12 h. The beads were then collected and boiled in 5× loading buffer for 10 min, and the supernatant was collected and subjected to SDS-PAGE for Western blotting and Coomassie blue staining. The corresponding band in the Coomassie blue stained gel matching anti-biotin immunoreactivity protein was excised manually and subjected to composition analysis using LC- ESI MS/MS analysis in Shanghai Applied Protein Technology Co. Ltd (Shanghai, China). The resulting MS/MS data were subjected to MASCOT search engine (http://www.matrixscience.com/, last accessed 19 June 2018) based on the UniProt database with the following parameter settings: trypsin-cleavage two missing cleavage sites allowed, carbamidomethyl (C) set as fixed modifications, oxidation (M) was allowed as variable modifications, and mass tolerances of precursor was 0.1 Da. Sequences of identified proteins were searched for using the BLAST server (https://blast.ncbi.nlm.nih.gov/Blast.cgi, last accessed 19 June 2018) to identify homologous sequences of *R*. *anatipestifer* Yb2 and putative functions.

### Density gradient centrifugation

Percoll density gradients were conducted as per Patrick and Reid^29^. Briefly, a stock solution of Percoll (Sigma Aldrich, St. Louis, MO, USA) was prepared by dilution with 1.5 mol NaCl at a ratio of 9:1(v/v). Solutions containing 80%, 70%, 60%, 50%, 40%, 30%, 20% and 10% Percoll in 0.15 mol NaCl were further prepared from the stock solution. A 1.2-ml volume of each of these solutions was carefully layered into a 13.2 ml polycarbonate tube to produce a step gradient with 80% Percoll at the bottom and the 10% Percoll at the top. One milliliter of each bacterial culture (OD_600_ = 2) suspended in 0.15 mol NaCl was applied to the top of the 10% layer and the gradient was then centrifuged for 1 h at 10,000 × g at 4 °C in a SW41Ti rotor using a Beckman centrifuge (Optima L-100XP, Beckman Coulter, Inc. CA, USA). The gradient was visualized using fuchsine stained layers as the marker (M).

### Biofilm formation assay

*R*. *anatipestifer* biofilm formation on borosilicate glass was measured by Live/dead BacLight Bacterial Viability staining as described previously^27^. Briefly, each bacterial culture in mid-exponential phase was adjusted to OD_600_ = 0.1 with TSB, and 1 ml of the bacterial suspension was transferred into 24-well polystyrene microtiter plates (Corning, NY, USA) containing sterile glass coverslips. The plates were incubated at 37 °C under an atmosphere of 5% CO_2_ and the coverslips were collected at 24 h and 48 h, respectively. After rinsing gently three times with sterile 0.01 mol PBS (pH7.2), the coverslips were stained with 100 μl of Live/dead BacLight Bacterial Viability staining reagent (Thermo Fisher Scientific, Waltham, MA, USA) for 15 min as according to the manufacture’s protocol, and examined using a fluorescence microscope (Nikon Eclipse 80i, Japan) in the dark at room temperature. The image profiles of bacterial shapes were visualized and analyzed using NIS-Elements Viewer software.

### Adhesion and invasion capacities to duck embryo fibroblast

Duck embryo fibroblast cells were used to evaluate the effects of the *R*. *anatipestifer* AS87_RS09170 gene on bacterial adherence and invasion. The cells were isolated from a 12-day-old duck embryo using standard conditions and then maintained at 37°C in a 5% CO_2_ atmosphere in Dulbecco’s Modified Eagle Medium (DMEM) (HyClone; GE Healthcare, Little Chalfont, UK), supplemented with 10% fetal bovine serum (Gibco; Thermo Fisher Scientific, Waltham, MA, USA). Prior to infection, duck embryo fibroblast cells were seeded (3 × 10^5^ cell per well) into 24-well tissue culture plates for 24 h. The cell monolayer was washed three times with sterile PBS and then infected with WT strain Yb2, mutant strain Yb2ΔbioF and complemented strain cYb2ΔbioF at 100 multiplicity of infection (MOI), respectively. The infected cells were incubated for 1.5 h at 37 °C in 5% CO_2_, washed three times with sterile PBS, and then lysed with 0.1% trypsin (100 μl/well). Serial dilutions of the cell suspensions were plated onto TSA plates to determine the numbers of viable bacterial cells. For the invasion assay, the extra-cellular bacteria were killed by incubating the infected cells with 100 μl/ml gentamicin for an additional 1 h. After washing three times with sterile PBS, the infected cells were lysed and the numbers of intracellular bacteria were enumerated by plating on a TSA plate. All above assays were performed in triplicate and replicated three times.

### Scanning and transmission electron microscope

The WT strain Yb2, mutant strain Yb2ΔbioF and complemented strain cYb2ΔbioF were grown to mid-exponential phase and harvested by centrifugation. The pellets were fixed in 2.5% phosphate-buffered glutaraldehyde (pH 7.2) for 24 h and post-fixed in 1% osmic acid. Dehydration was accomplished by a graded series of acetone. For scanning electron microscopy, the samples were critical-point dried and sputter coated with a thin layer of gold. Photographs were taken using a Tecnai G2 F30 scanning electron microscope (FEI, Hillsboro, OR, USA). For transmission electron microscopy, the samples were fixed, post-fixed and dehydrated under the same conditions described above. Epoxy resin was then infiltrated into the fixed and dehydrated samples and polymerized into a plastic block. Subsequently, the block was sliced into ultra-thin sections and stained with both uranyl acetate and lead citrate. The specimens were observed on a Tecnai 12 transmission electron microscope (FEI, Eindhoven, Netherlands) at 80 kV and images were recorded using Ditabis imaging plates.

### Extraction and identification of membrane proteins

Membrane proteins were isolated from *R*. *anatipestifer* WT strain Yb2, mutant strain Yb2ΔbioF and complemented strain cYb2ΔbioF, respectively, using a bacterial membrane protein extraction kit (BestBio, Shanghai, China) according to the manufacturer’s protocol. Briefly, *R*. *anatipestifer* strains were cultured respectively to logarithmic phase (OD_600_ = 1.5), the bacterial pellets were collected and washed twice by centrifugation. The extract buffer was then prepared and added to the bacterial pellets, the mixture was stirred for 2 h at 4 °C to lyse bacteria. The supernatant was collected by centrifugation for 15 min at 4 °C and incubated at 37 °C in water-bath for 30 min for stratification. After the upper layer liquid was removed, menbrane protein dissolution buffer was added to make the membrane proteins. The proteins were separated by SDS-PAGE and stained with Coomassie blue. The differentially expressed protein band in the mutant strain Yb2ΔbioF was excised manually from the gel and subjected to LC-ESI-MS/MS analysis in Shanghai Applied Protein Technology Co. Ltd (Shanghai, China) as described above, and searched for using the online software PSORTb version 3.0.2 to predict the subcellular location of each protein^30^.

### Illumina sequencing for RNA-Seq and differential expression analysis

The WT strain Yb2, mutant strain Yb2ΔbioF and complemented strain cYb2ΔbioF were cultured in TSB medium at 37 °C with shaking for 12 h. The total RNA was extracted with TRIzol regent (Invitrogen) according to the manufacturer’s instructions. Total RNA quantity and quality were assessed by Agilent 2100 Bioanalyzer (Agilent RNA 600 Nano kit) and ribosome RNAs were depleted using Ribo-Zero Magnetic Gold Kit (epicenter, USA). The illumina RNA-Seq libraries were generated and then validated by the Agilent 2100 bioanalyzer instrument (Agilent DNA 1000 Reagents) and real-time quantitative PCR (qPCR) (TaqMan Probe). The qualified libraries were amplified on cBot to generate the cluster on the flowcell (TruSeq PE Cluster Kit V3-cBot-HS, Illumina), and the amplified flowcell was sequenced pair end on the HiSeq 2000 System (TruSeq SBS KIT-HS V3, Illumina)^31^. Low-quality reads and adaptors were removed from raw reads. Cleaned reads were aligned to the *R*. *anatipestifer* Yb2 genome using RNA Sequel software HISAT (Version 2.0.1-beta)^32,33^. Transcript levels were calculated as RPKM (Reads per kilobase cDNA per million fragments mapped) using RSEM software (version 1.2.12)^34^. Differentially expressed genes were analyzed using possionDis with fold change (cutoff = 2.0)^35,36^, and considered statistically significant if the fold change was >2.0 and the FDR (False Discovery Rate) was <0.05.

### Real-time quantitative PCR analysis

qPCR was performed to confirm transcriptional levels of differentially expressed genes obtained in the RNA-Seq analysis. Gene-specific primers were designed using primer3 online software Version.0.4.0^37^ and are described in supplementary Table S1. The expression of the L-lactate dehydrogenase encoding gene (ldh) was measured using primers RA ldh-F/RA ldh-R, and used as an internal control. Total RNA was isolated from the WT strain Yb2 and mutant strain Yb2ΔbioF using Trizol reagent (Invitrogen, Carlsbad, CA, USA), according to the manufacturer’s instructions. All RNA samples were treated with the TURBO DNA-free kit (Ambion, Grand Island, NY, USA) to remove DNA contamination. cDNA was synthesized using PrimeScript RT Master Mix (Takara). qPCR was conducted in Go Taq qPCR Master Mix (Promega, Fitchburg, WI, USA) using the following parameters: 95 °C for 2 min, 40 cycles of 95 °C for 15 s, 55 °C for 15 s and 68 °C for 20 s, followed by one cycle of 95 °C for 15 s, 60 °C for 15 s and 95 °C for 15 s. Reactions were performed in triplicate and run on the Mastercycler ep realplex4 apparatus (Eppendorf, Germany). Quantification of transcriptional level was calculated according to the 2^−ΔΔCt^ method.

### Bacterial virulence determination

The bacterial loadings in the blood of infected ducks were counted to evaluate bacterial survival *in vivo*^38^. Eighteen-day-old ducks (six ducks per group) were inoculated intramuscularly with the WT strain Yb2 and mutant strain Yb2ΔbioF at 2.5 × 10^8^ CFU in 0.5 ml PBS. The blood samples were collected at 12 h, 24 h and 36 h post infection, diluted appropriately and plated on TSA for bacterial counting^25^.

### Statistical analysis

Statistical analyses were conducted using GraphPad Software version 6.0 (La Jolla, CA, USA). One-way analysis of variance (ANOVA) was used for analyses of growth curves, adhesion and invasion data; and the two-tailed independent Student’s *t* test was performed for analyses of bacterial loads in blood. Statistical significance was established at *p* < 0.05.

## Electronic supplementary material

Supplementary Information
