# Supplementary material for: Riemerella anatipestifer AS87_RS09170 gene is responsible for biotin synthesis, bacterial morphology and virulence
Source: Sci Rep. 2018 Oct 2;8:14645. doi: 10.1038/s41598-018-32905-1 (PMC6168606; doi:10.1038/s41598-018-32905-1)
Supplement: Supplementary file 1 — Supplementary Information [file 41598_2018_32905_MOESM1_ESM.pdf]

Supplementary Information

Riemerella anatipestifer AS87_RS09170 gene is responsible for biotin synthesis, bacterial morphology and virulence

Xiaomei Ren[1,※], Xiaolan Wang[1,※], Huoying Shi[2], Xuemei Zhang[1], Zongchao Chen[1], Kanwar Kumar Malhi[1], Chan Ding[1,3] and Shengqing Yu[1,3*]

[1]Shanghai Veterinary Research Institute, Chinese Academy of Agricultural Sciences (CAAS), Shanghai, China

[2]College of Veterinary Medicine, Yangzhou University, Yangzhou, Jiangsu, China

[3]Jiangsu Co-innovation Center for Prevention and Control of Important Animal Infectious Diseases and Zoonosis, Yangzhou, Jiangsu, China

Corresponding authors: Prof Shengqing Yu, E-mail: yus@shvri.ac.cn.

※X.R. and X.W. contributed equally to this work.

Supplementary Figure S1. The putative biotin biosynthetic pathway in *R. anatipestifer*. Pimeloyl-[acp] was synthesized from malonyl-CoA through the fatty acid biosynthetic pathway in *R. anatipestifer*. The subsequent four steps utilizing BioF, BioA, BioD and BioB for biotin synthesis are conserved in all organisms. BioF can accept either pimeloyl-[acp] or pimeloyl-CoA as a substrate. This pathway is proposed to exist in *R. anatipestifer* due to the presence of putative FabD, FabF, FabG, FabZ and FabI, and absence of BioI and BioW homologues in the *R. anatipestifer* genome. Abbreviations: CoA, Coenzyme A; acp, acyl carrier protein; FabD, [acp] S-malonyltransferase; FabF, 3-oxoacyl-[acp] synthase II; FabG; 3-oxoacyl-[acp] reductase; FabZ, 3-hydroxyacyl-[acp] dehydratase; FabI, enoyl-[acp] reductase; BioF, 8-amino-7-oxononanoate synthase; BioA, adenosylmethionine-8-amino-7-oxononanoate aminotransferase; BioD, dethiobiotin synthetase; BioB, biotin synthase.

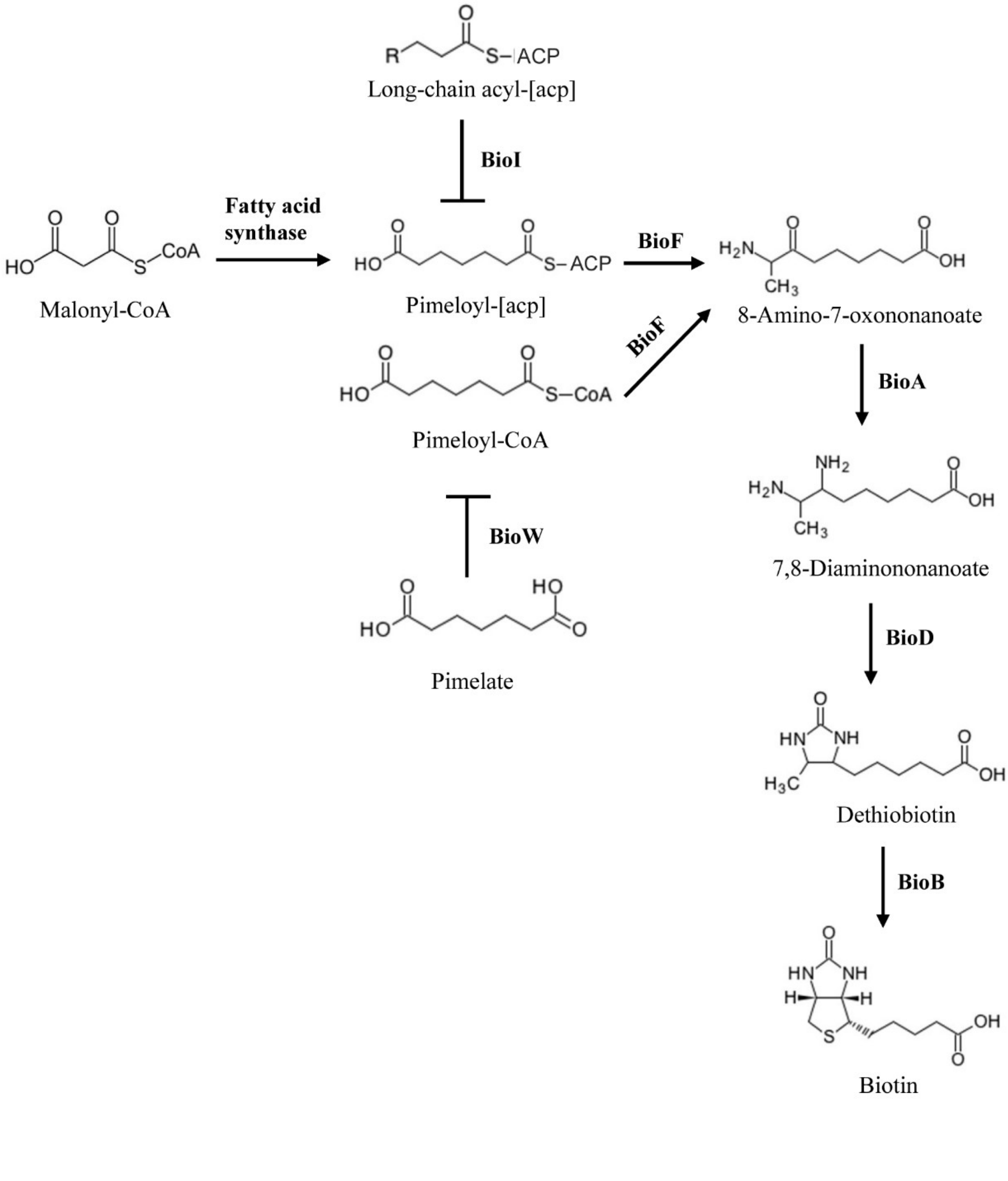

Supplementary Figure S2. The uncropped image of PCR analyses of *R. anatipestifer* AS87_RS09170 gene. The cropped image was labeled in red frame.

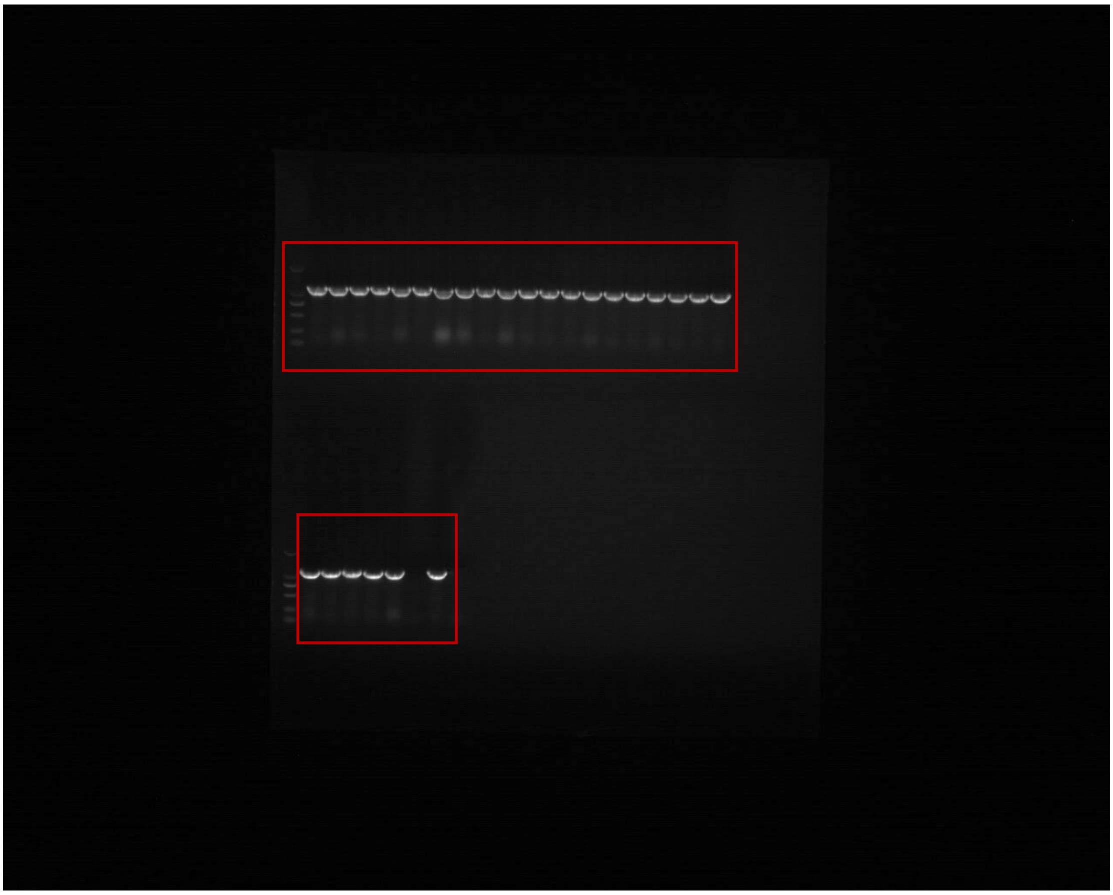

Supplementary Figure S3. Immunoblot analysis of the biotinylated protein. The cropped blots correspond to GroEL were labeled in red frame of row A; the cropped blots correspond to biotinylated proteins were labeled in red frame of row B.

A

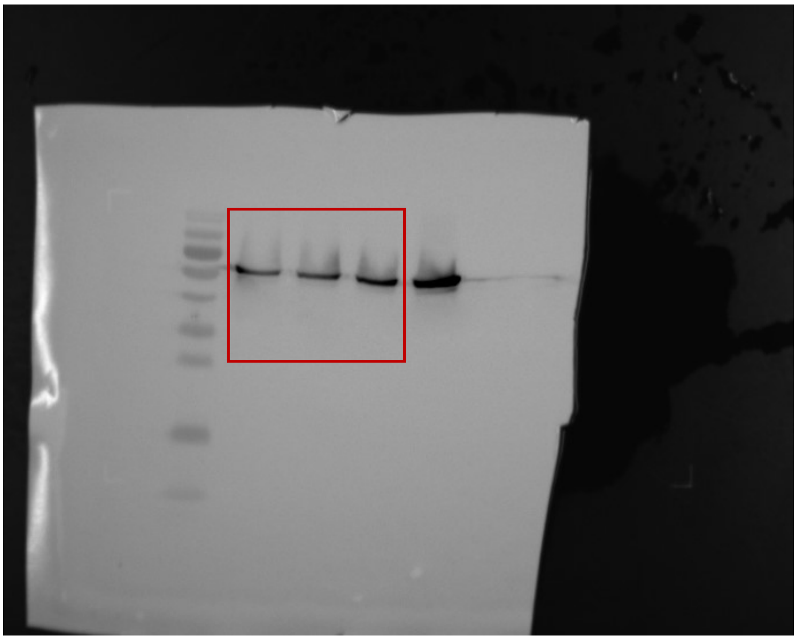

B

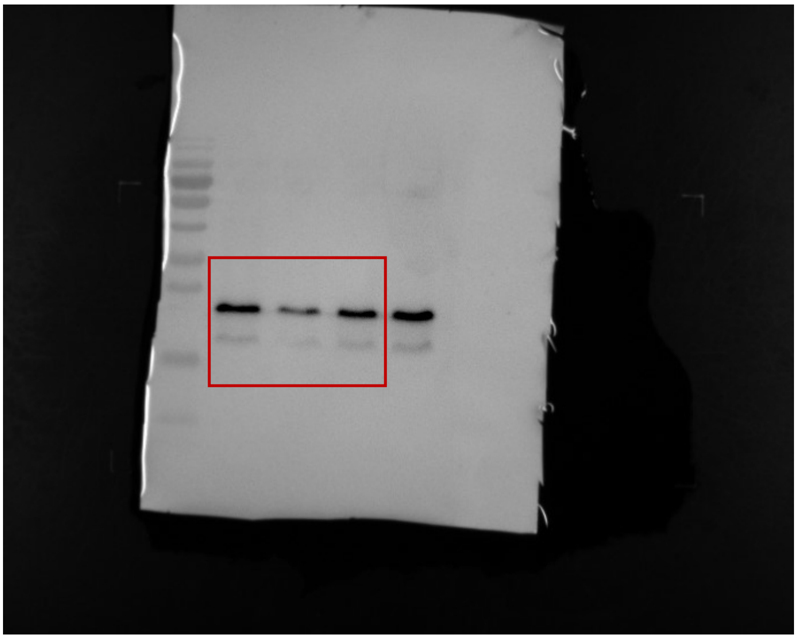

Supplementary Figure S4. Analysis of membrane proteins. The cropped gel was labeled in red box.

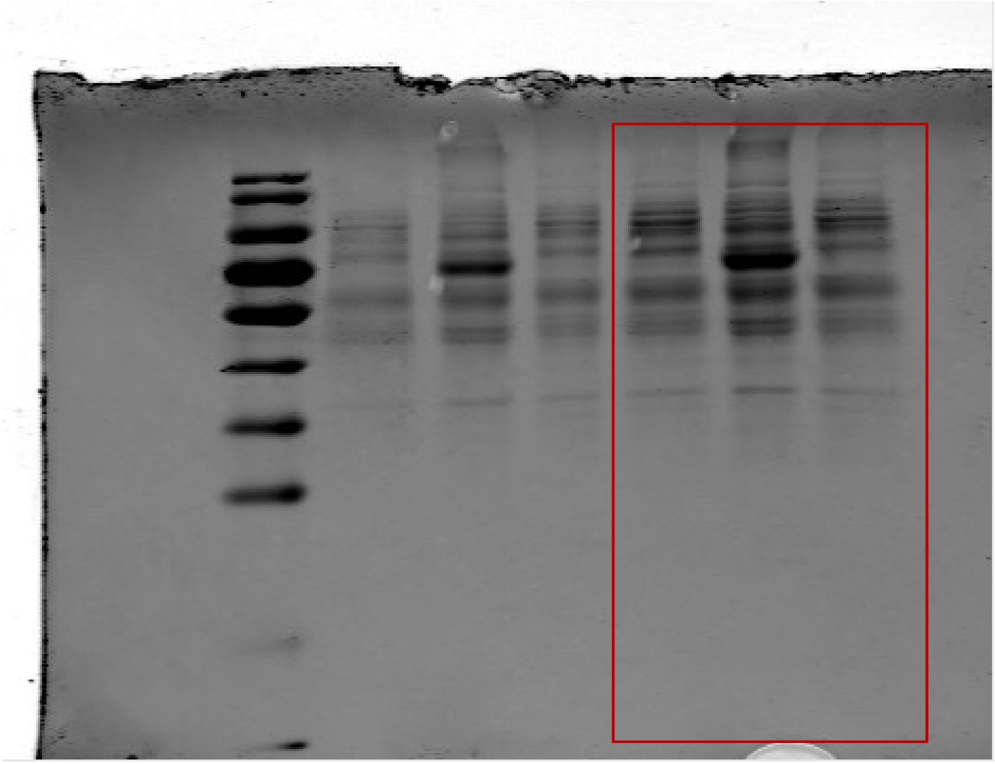

Supplementary Table S1. Primers used for qPCR analysis

Gene locus[a]	Forward primer sequences (5' - 3')	Reverse primer sequences (5' - 3')	Products (bp)
AS87_RS05800	TTACAACGCATTGAAAGCAGA	TGGCACTATAAGCTGTGAGGAA	184
AS87_RS04150	CGTGCTAAGAGCTACGATGG	GGGCGAAGATCTCCTTTTTC	183
AS87_RS04120	AATGATGGATGGAGCCAAAG	CAATCCTGAGTCAAATTCCGTA	434
AS87_RS09460	GCATCCTGCAGTTGCTTTTA	TTCTGCTTTTCTTGGGTGCT	244
AS87_RS09445	GCATCCTGCAGTTGCTTTTA	TTCTGCTTTTCTTGGGTGCT	244
AS87_RS03745	TTTGGGTCCTAGGCTGAATG	AAAAGCCTTCTACCCCAAACTC	194
AS87_RS05725	TCTCCGCGACTTTGTTTTCT	GTCGCTACCATCTGGGTGAT	179
AS87_RS05655	AGATGGAAGTGGCATTGCTC	CTTGCTCCTCAAAACCTTCG	230
AS87_RS02380	CGTATCATTCGGAACGCTCT	AGGGACAATGTTGGCTTTTG	197
AS87_RS06190	CATTATCGCCCTAGCAAAGC	TTACCAGAAGCGGTGTCTCC	216
AS87_RS03960	AAAAGGAATCCACCCTGAAAA	CCAGTGTAGAATGGGTGAGAAG	174
AS87_RS01395	AAAACTTTGGGGGCAATTCT	GATAACAGCGTTCGGCATTT	241
AS87_RS10270	GCGGACAAAATTCTCCCTTT	ATCCGAAGTCCAAGCATTTG	183
AS87_RS02825	CCGAGGACGAAAACCATTTA	TGGCTACTTGCTGTGCAAAC	150
AS87_RS07030	GGAGAAAGACCTTGGGGAAA	TTTGAGCCAAGGGGTATGAG	218
AS87_RS10115	TTTGGGACGATAAACATTTTCC	TTTTCAAGGTGGGAGATGATG	154
AS87_RS01890	AACGGTCTTGCAGTGGTAGG	TCTTCAATCACCAAATCGTCA	158
AS87_RS10350	AGCTCTACCTCCGTTCACCA	TTCCTTCTCATTGGTGCATCT	159-
AS87_RS06890	GCGTTTGGGCAGGATATAGA	AAAAGTGCATCTCCTCCTTCA	161
AS87_RS02260	AGGTGCCAGAGTTTGAGCAT	TCGTCCCATAGTTCCTGTCC	172
AS87_RS01190	TGGAAAATCACATTTTAATCAACG	TGGTTTTGCTCTCCTGCTTC	151
AS87_RS00965	TCCCTGAATTCAAAGCTGGT	CATCGCCACTCATTTTTCTG	153
AS87_RS05265	CGAGCAAATAAATGCGGAAA	CGGAAATAAACTTCGCCATC	189
AS87_RS09430	ATGGAGCGGAAAAACACATT	CACCCATTGGTAAAGGGAAA	150
AS87_RS07650	ATGGCAAATCATAAATCAGCA	CTTAGGCAATTGCTCTGATGC	150
AS87_RS00160	TAGTGGGCGGTTTTTCAATC	GCCTCCTTGTATGCTTTTGC	229
AS87_RS08680	TACCACAGGAGCACAGCAAG	AAGCGTTGGCTTCATACGAT	239
AS87_RS02480	GGTTTTGGCATTTCCCTTTT	CGGGTTTTATACGCTCCAAA	161
AS87_RS09175	AGAGGCTTTAGGAGCCGATT	CACACCACCTGCACCTTCTA	244
AS87_RS01370	GAGCAGCTGTATTGGGTGCT	TATTCTCCCGCTGCCATAGA	151
AS87_RS07305	AAAACCGGGGGAAACAGTAG	CCACTCAGGCACTGGAGTTT	220
AS87_RS01365	GGGTTAGTGCGATTTGTGGT	CTCCCAAAAAGACAGCAGGA	207
AS87_RS01360	GGAGCTTCGTTTTGTCTTGC	CCTTGTGGCGGAACATCTAT	196
AS87_RS01355	CGCAGAAAACTTTAAGGCACA	GCAAGTCTAGCCATTTCCACA	234
AS87_RS01350	CGAAGTTTTTCTCGCTTTGG	TAACGGTGCTCGTAGCCTTT	218
AS87_RS10025	ATCACCCAAGCGATTGAAAG	TGGATTTGGCTCAGGATTTC	186
AS87_RS07820	TACCACTTCCGATGGCTACC	TCCCCATAGCCTCATACTGG	238
AS87_RS09180	GTTTTGGCGGAAACGATTTA	CACCGCCGTAGAACCATTAT	162
AS87_RS09185	GCGGTGGAAAAGCAGTATTC	GCATCTAAAAGCCCTCCTGTT	164

[a] Based on *R. anatipestifer* Yb2 genome (accession number: CP007204).

Supplementary Table S2 Different expressed protein of mutant strain Yb2ΔbioF identified by Liquid Chromatography (LC)-Electrospray Ionization (ESI) Tandem MS (MS/MS)

Accession no.	Protein name	Subcellular location[a]
AKQ39270	endothelin-converting protein	Cytoplasmic
AKQ40024	peptidase S46	Cytoplasmic
AKQ39536	succinate dehydrogenase	Cytoplasmic Membrane
AKQ40081	ABC transporter ATP-binding protein	Cytoplasmic
AKQ38971	threonyl-tRNA synthetase	Cytoplasmic
AKQ38866	peptidase S8	Cytoplasmic
AKQ39160	glutamate--tRNA ligase	Cytoplasmic
AKQ40412	hypothetical protein AS87_08865	Cytoplasmic
AKQ40494	pyruvate dehydrogenase	Cytoplasmic
AKQ39187	molecular chaperone DnaK	Cytoplasmic
AKQ40079	hypothetical protein	Cytoplasmic
AKQ39439	phosphoglucomutase	Cytoplasmic
AKQ38925	asparagine synthetase B	Cytoplasmic
AKQ40192	urease subunit alpha	Cytoplasmic
AKQ38988	GTP-binding protein TypA	Cytoplasmic
AKQ39014	30S ribosomal protein S1	Cytoplasmic
AKQ40083	membrane protein	Outer Membrane
AKQ39944	ribonuclease R	Cytoplasmic
AKQ39296	peptidase M3	Cytoplasmic
AKQ39451	Xaa-Pro aminopeptidase	Cytoplasmic
AKQ39970	aminopeptidase	Unknown
AKQ39114	peptidase S46	Cytoplasmic
AKQ39755	TonB-dependent receptor	Outer Membrane
AKQ39984	elongation factor 4	Cytoplasmic Membrane
AKQ39047	DNA gyrase subunit B	Cytoplasmic
AKQ39656	glycoside hydrolase	Cytoplasmic
AKQ40119	peptidylprolyl isomerase	Periplasmic
AKQ39883	peptidase M41	Cytoplasmic Membrane
AKQ39061	2-oxoglutarate dehydrogenase	Cytoplasmic
AKQ40395	glutamine amidotransferase	Cytoplasmic
AKQ38983	lysyl-tRNA synthetase	Cytoplasmic
AKQ40550	aconitate hydratase	Cytoplasmic
AKQ39348	polynucleotide phosphorylase	Cytoplasmic
AKQ39527	arginyl-tRNA synthetase	Cytoplasmic
AKQ40394	hypothetical protein AS87_08775	Outer Membrane
AKQ39033	dipeptidyl carboxypeptidase II	Cytoplasmic
AKQ39771	preprotein translocase subunit SecA	Cytoplasmic

AKQ39501	asparagine synthase	Cytoplasmic
AKQ39124	AMP-dependent synthetase	Cytoplasmic Membrane
AKQ40011	tRNA uridine 5-carboxymethylaminomethyl modification protein	Cytoplasmic
AKQ39642	malic enzyme	Cytoplasmic
AKQ40166	hypothetical protein AS87_07530	Unknown
AKQ40159	leucyl-tRNA synthetase	Cytoplasmic
AKQ38946	formate--tetrahydrofolate ligase	Cytoplasmic
AKQ39828	TonB-dependent receptor	Unknown
AKQ39103	phenylalanyl-tRNA synthetase subunit beta	Cytoplasmic
AKQ39505	asparagine synthase	Cytoplasmic
AKQ38848	ATP-dependent DNA helicase RecQ	Cytoplasmic
AKQ39796	DNA replication protein RecF	Cytoplasmic
AKQ38936	peptidase M16	Unknown
AKQ39988	isoleucyl-tRNA synthase	Cytoplasmic
AKQ39112	transketolase	Cytoplasmic
AKQ40669	hypothetical protein AS87_10250	Unknown
AKQ39605	elongation factor Tu	Cytoplasmic
AKQ39119	methionyl-tRNA synthetase	Cytoplasmic
AKQ38954	DNA topoisomerase IV subunit B	Cytoplasmic
AKQ39138	2-oxoglutarate dehydrogenase	Cytoplasmic
AKQ39812	translation initiation factor IF-2	Cytoplasmic
AKQ38954	DNA topoisomerase IV subunit B	Cytoplasmic
AKQ38924	aspartyl-tRNA synthetase	Cytoplasmic
AKQ39593	DNA-directed RNA polymerase subunit beta	Cytoplasmic
AKQ38911	TonB-dependent receptor	Outer Membrane
AKQ39740	fumarate hydratase	Cytoplasmic
AKQ39940	Clp protease ClpC	Cytoplasmic
AKQ38789	catalase	Unknown
AKQ39732	peroxidase	Cytoplasmic
AKQ40305	alanyl-tRNA synthetase	Cytoplasmic
AKQ38951	ribonuclease	Cytoplasmic
AKQ39298	membrane protein	Outer Membrane
AKQ39918	quinol:cytochrome C oxidoreductase	Unknown
AKQ40246	peptidase M16	Outer Membrane
AKQ39589	ABC transporter	Cytoplasmic
AKQ40473	peptidase S9	Unknown
AKQ39688	catalase	Periplasmic
AKQ39388	hypothetical protein AS87_03385	Cytoplasmic
AKQ39618	bifunctional cbb3-type cytochrome c oxidase subunit I/II	Cytoplasmic
AKQ39926	peptidoglycan glycosyltransferase	Cytoplasmic Membrane

AKQ39592	DNA-directed RNA polymerase subunit beta'	Cytoplasmic
AKQ38812	acyl-CoA dehydrogenase	Cytoplasmic
AKQ39486	prolyl tripeptidyl peptidase	Unknown
AKQ39444	valyl-tRNA synthetase	Cytoplasmic
AKQ40043	hypothetical protein	Cytoplasmic
AKQ39981	cysteine desulfurase	Cytoplasmic
AKQ39198	dihydrolipoamide dehydrogenase	Cytoplasmic
AKQ39125	peptide chain release factor 3	Cytoplasmic
AKQ38846	gliding motility protein GldG	Unknown
AKQ39839	glutamine synthetase	Cytoplasmic
AKQ39153	hypothetical protein	Outer Membrane
AKQ39462	glycine dehydrogenase	Cytoplasmic
AKQ39948	TonB-dependent receptor	Outer Membrane
AKQ39968	histidine kinase	Unknown
AKQ39809	hypothetical protein	Outer Membrane
AKQ39137	2-oxoglutarate dehydrogenase	Cytoplasmic
AKQ40290	ribonucleotide-diphosphate reductase subunit alpha	Cytoplasmic
AKQ38865	RNA polymerase sigma24 factor	Cytoplasmic
AKQ40353	hypothetical protein	Cytoplasmic Membrane
AKQ40201	gliding motility protein	Outer Membrane
AKQ38943	phosphate starvation protein PhoH	Cytoplasmic
AKQ38978	phosphoribosylformylglycinamidine synthase	Cytoplasmic
AKQ39310	DNA mismatch repair protein MutS	Cytoplasmic
AKQ40244	recombinase RecJ	Cytoplasmic
AKQ40435	hypothetical protein AS87_08980	Cytoplasmic
AKQ38939	DNA polymerase I	Cytoplasmic
AKQ39232	5-methyltetrahydropteroyltriglutamate--homocysteine methyltransferase	Cytoplasmic
AKQ40583	hypothetical protein	Cytoplasmic
AKQ40066	phospho-N-acetylmuramoyl-pentapeptide-transferase	Cytoplasmic Membrane
AKQ39325	NifR3 family TIM-barrel protein	Cytoplasmic
AKQ39331	peptidase M1	Cytoplasmic
AKQ40443	serine dehydratase	Cytoplasmic
AKQ38958	Na(+)/H(+) antiporter NhaA	Cytoplasmic Membrane
AKQ38976	3-hydroxybutyryl-CoA dehydrogenase	Cytoplasmic
AKQ39903	protease 2	Periplasmic
AKQ39452	1-pyrroline-5-carboxylate dehydrogenase	Cytoplasmic
AKQ40265	Clp protease ClpB	Cytoplasmic
AKQ39635	hypothetical protein AS87_04735	Cytoplasmic Membrane
AKQ40379	translation factor Sua5	Unknown

AKQ39516	glucose-6-phosphate isomerase	Cytoplasmic
AKQ39843	histidine kinase	Cytoplasmic Membrane
AKQ39612	pseudouridine synthase	Cytoplasmic
AKQ39862	excinuclease ABC subunit B	Cytoplasmic
AKQ39736	glycyl-tRNA synthetease	Cytoplasmic
AKQ39010	phosphoribosylaminoimidazolecarboxamide formyltransferase	Cytoplasmic
AKQ38930	membrane protein	Cytoplasmic Membrane
AKQ39100	molecular chaperone GroEL	Cytoplasmic
AKQ38932	isocitrate dehydrogenase	Cytoplasmic
AKQ39532	CTP synthetase	Cytoplasmic
AKQ39807	amino acid ABC transporter substrate-binding protein	Cytoplasmic
AKQ39086	preprotein translocase subunit SecD	Cytoplasmic Membrane
AKQ40007	phosphoenolpyruvate carboxykinase	Cytoplasmic
AKQ39811	TonB-dependent receptor	Outer Membrane
AKQ39719	elongation factor G	Cytoplasmic
AKQ38777	alpha-glucosidase	Unknown
AKQ40273	prolyl endopeptidase	Periplasmic

[a]Subcellular locations were predicted by the PSORTb v.3.0.2 software (http://www.psort.org/).
